# Prevalence of living wills among older adults in Germany

**DOI:** 10.25646/11665

**Published:** 2023-09-20

**Authors:** Susanne Wurm, Svenja M. Spuling, Ann-Kristin Reinhard, Ulrike Ehrlich

**Affiliations:** 1 University Medicine Greifswald, Department Prevention Research and Social Medicine, Germany; 2 German Centre of Gerontology, Berlin, Germany

**Keywords:** PREVENTION, PREVALENCE, LIVING WILLS, GERMAN AGEING SURVEY 2020/2021, MIDDLE AGED, AGED

## Abstract

**Background:**

Living wills regulate medical decisions in emergency situations. Those who create a living will can have it registered voluntarily in the Central Register of Lasting Powers of Attorney. Little is known about the general prevalence of living wills.

**Methods:**

The German Ageing Survey is an ongoing, population-representative study. 4,185 people aged 50 and older were surveyed about living wills in 2020/2021.

**Results:**

44.8 % of people aged 50 and older have a living will, women more often than men (50.1 % vs. 39.2 %), older people more often than middle-aged people. Educational differences do not exist.

**Conclusions:**

Living wills increase the autonomy in medical emergency situations because the patient’s wishes are specified in written form. People of all age groups should inform themselves about the significance of living wills and should seek advice about the contents, for example from the general practitioner or one’s own health insurance.

## Introduction

Suddenly due to an accident or gradually due to a chronic illness – all people, whether at younger or older ages, can find themselves in a medical emergency situation, in which they are no longer able to communicate their own wishes or to make decisions. In that case, matters can be governed preventively in Germany. While a healthcare proxy or care decree transfers the rights for personal matters (e.g., finances, contacts, etc.) to another person, a living will governs *medical decisions* in emergency situations.

In a living will, any adult person can record in written form, which medical and care provisions they want or deny in the concrete case of certain health conditions (e.g., after brain damage or in the final stage of an incurable, fatal illness), be it examinations, curative treatments, or medical interventions. It can be determined, for example, whether artificial feeding is to take place, which pain medication is requested, or how one feels about organ donation. The Federal Ministry of Justice additionally suggests specifying further values in the living will, e.g., religious beliefs or attitudes about life and death. When preparing a catalogue of measures, it is suggested to seek guidance from a physician. If the documented patient’s wishes are worded in a way that concrete medical situations and measures are addressed clearly, the attending physicians are obligated to follow these wishes. A living will has to be written autonomously and becomes valid by means of personal signature [1]. The notarization of the living will by a notary as well as an official registration in the Central Register of Lasting Powers of Attorney (ZVR) is possible but not mandatory [2, 3]. Until January 1, 2023, living wills could only be registered in the ZVR as part of an advance directive. In the past year, a total of approx. 5.7 million advance directives were registered, which continues the increase of registered directives of previous years [4–6]. Of the newly registered advance directives in 2022, 77.1 % were combined with a living will. Because there is no obligation to report, however, the ZVR can only hint at the prevalence of living wills [6].

There are only few comprehensive studies relating to the prevalence of living wills in Germany. The collected survey data thereby often originate from selective subpopulations (e.g., patients) and are not representative for the general population. A telephone survey of the German Hospice and Palliative Association (DHPV) among around 1,000 people aged 18 and older showed, e.g., that 43 % of the respondents in Germany have a living will [7]. In another study with around 1,000 people, it became clear that a living will more often existed among patients in intensive care units of a university hospital, who had elective (i.e., planned) surgeries, than among those who required emergency surgery [8]. As reasons for a living will, the survey participants indicated, for example, being afraid of being dependent on the decisions of others, of no longer having autonomy, or of not wanting to get over-treatment.

So far, the ZVR and the research literature does not provide a clear data situation with regard to the prevalence of living wills. In contrast, the present study analyses the current prevalence of living wills among women and men in different age and education groups of the population aged 50 and older on the basis of nationwide representative data from the German Ageing Survey (DEAS).

### Indicator

The existence of a living will was captured in the German Ageing Survey (DEAS) 2020/2021 by self-reported information from respondents in a questionnaire filled out in paper form or online. The DEAS is a nationwide representative cross-sectional and longitudinal survey of people who are in the second half of life and are thus at least 40 years of age. The first survey took place in 1996, and six follow-ups have taken place since then. In the 2020/2021 survey year, 5,402 people between the ages of 46 and 100 participated in the oral interview; 4,419 of these respondents (82 %) also completed the additional questionnaire. The questionnaire contained the question: ‘Have you made one or several of the following written instructions or legal arrangements?’. Among others, the existence of a living will was assessed. The respondents had three response options: ‘yes’, ‘no’, ‘don’t know what that is’. Respondents that selected the response category ‘don’t know what that is’ (12 respondents: 4 women, 8 men) were assigned to the response category ‘no’. Respondents with missing information about the existence of a living will (98 respondents: 56 women, 42 men) as well as respondents younger than 50 years of age (105 respondents: 55 women, 50 men) or older than 90 years of age (31 respondents: 12: women, 19 men) were excluded. The analysis sample thus consisted of 4,185 respondents between the ages of 50 and 90 (2,134 women, 2,051 men). The 1997 International Standard Classification of Education (ISCED) was used to classify respondents’ educational and vocational attainments [9]. Due to a small number of cases with low education level, the groups of people with low and medium education were combined. Weighted prevalences as percentages with 95 % confidence intervals (95 % CI) were presented on the presence of living wills stratified by gender, age and education using methodology that takes into account the stratified sampling of the DEAS. Descriptive results with the respective confidence intervals are presented in tabular form. In addition, a significance test was conducted to test for differences between the groups. A detailed description of the DEAS methodology is presented elsewhere [10, 11].

## Results and conclusion

In total, 44.8 % of respondents report having a living will. There are statistically significant gender differences: 50.1 % of women, but only 39.2 % of men indicate having a living will. Among both genders, the prevalence of living wills increases significantly across age groups (Table 1). While 31.7 % of the women aged 50 to 64 indicate having a living will, already 61.5 % among those aged 65 to 74 indicate having one. Among women aged 75 and older, more than three of four women (76.5 %) have a living will. With 28.9 %, men between the ages of 50 and 64 have a living will significantly far less often than the group of the 65- to 74-year-olds (39.6 %); among men, the highest prevalence can also be found in the age group of those aged 75 and older (68.5 %). However, whether or not a living will exists does not depend in a statistically significant way on the educational background, neither among women nor among men (Table 1).

While a registration of the living will in the ZVR is voluntary and thus selective, self-reported information from respondents of a random sample provide a potentially more comprehensive picture about the prevalence of living wills in Germany. The nationwide representative data of the DEAS uncover that even during the COVID-19 pandemic period 2020/2021, less than half of all people aged 50 and older report having a living will. Between the ages of 50 and 64, only every fourth person has a living will; however, across age groups, the prevalence of having a living will partially increases significantly. Additional comparisons with the DEAS from 2017 do not show an increase in the prevalence of living wills over the last three to four years (44.7 % in 2017 compared to 44.8 % in 2020/2021).

The availability and interpretability of the expressed preferences in a patient’s living will are extremely important for medical treatments and emergency situations. Since January 1, 2023, treating physicians can access information from the ZVR, if a person is unable to respond and if an urgent medical decision has to be made [6]. However, if a living will does not exist, if it cannot be found or contains contradictory wording, the patient’s assumed will has to be determined and interpreted [1, 2]. To clearly determine the patient’s assumed will, the living will can be supplemented with a healthcare proxy, which designates an authorized person for this case [1]. According to data from the DEAS, the majority of people with a living will also have a healthcare proxy (84.9 %). To inform about the importance of living wills and to counsel patients with regard to their personal preferences, general practitioners could have an important pilot function. However, the statutory health insurance currently does not pay for counselling about living wills.

## Key statement

- In 2020/2021, 50.1 % of women and 39.2 % of men aged 50 and older indicate having issued a living will.
- Compared to middle-aged people, older people issue a living will more frequently.
- Whether people have issued a living will is independent of the education level.

## Figures and Tables

**Table 1:** Prevalence of the existence of a living will by gender, age and education (n = 2,134 women, n = 2,051 men) Source: German Ageing Survey (2020/2021)

	%	(95 % CI)
**Women (total)**	**50.1**	**(45.0 – 55.2)**
**Age groups**		
50 – 64 years	31.7	(26.2 – 37.7)
65 – 74 years	61.5	(53.8 – 68.6)
≥ 75 years	76.5	(67.6 – 83.6)
**Education**		
Low/medium	48.7	(42.1 – 55.3)
High	53.5	(46.5 – 60.3)
**Men (total)**	**39.2**	**(34.8 – 43.8)**
**Age groups**		
50 – 64 years	28.9	(23.6 – 34.9)
65 – 74 years	39.6	(32.2 – 47.5)
≥ 75 years	68.5	(56.8 – 78.3)
**Education**		
Low/medium	36.7	(30.2 – 43.7)
High	41.9	(36.3 – 47.8)
**Total (women and men)**	**44.8**	**(41.2 – 48.5)**

CI = confidence interval
